# First Report of *Xiphinema Ifacolum* Luc, 1961 (Dorylaimida: Longidoridae) from Nigeria

**DOI:** 10.2478/jofnem-2022-0015

**Published:** 2022-06-17

**Authors:** Tesleem T. Bello, Oluwatoyin A. Fabiyi, Ilenia Clavero-Camacho, Carolina Cantalapiedra-Navarrete, Juan E. Palomares-Rius, Pablo Castillo, Antonio Archidona-Yuste

**Affiliations:** 1 Federal College of Education, Abeokuta, Ogun State, Nigeria; 2 Department of Crop Protection, Faculty of Agriculture, University of Ilorin, Ilorin, Kwara State, Nigeria; 3 Institute for Sustainable Agriculture (IAS), CSIC, Campus de Excelencia Internacional Agroalimentario, ceiA3, 14004 Córdoba, Spain; 4 Andalusian Institute of Agricultural and Fisheries Research and Training (IFAPA), Centro Alameda del Obispo, 14004 Córdoba, Spain

**Keywords:** Africa, Cytochrome oxidase c subunit 1, D2-D3 expansion segments of 28S rRNA gene, Dagger nematodes, Taxonomy

## Abstract

A population of a species of dagger nematode (*Xiphinema*) belonging to the non-*americanum* group was recovered from the fields of kola nut (*Cola* sp.) in southern Nigeria. The morphological and morphometric data obtained from this population were consistent with the characteristics of the species *Xiphinema ifacolum*. In addition, molecular identification based on D2-D3 expansion segments of 28S rRNA and partial mitochondrial *COI* gene regions confirmed its identity. According to our knowledge, this is the first report of the species from Nigeria, and the second report from Africa, after the original description from Foulaya, Guinea.

Dagger nematodes of the genus *Xiphinema* Cobb, 1913 are polyphagous root ectoparasites parasitizing a wide range of economically important plants by directly feeding on root cells. It has been demonstrated that some species of this genus trans-mit nepoviruses (Taylor and Brown, 1997). Particularized identification of the various member species of this genus is rendered difficult by the fact that they share various morphological characteristics, which show minimal variation. Also, the occurrence of complex cryptic species within *Xiphinema* (Pedram *et al*., 2012; Palomares-Rius *et al*., 2014; Archidona-Yuste *et al*., 2016a,b; Zhao *et al*., 2017; Cai *et al*., 2020) makes it necessary to apply integrative taxonomical approaches based on morphology and molecular markers. The non-*americanum*-group species of *Xiphinema* comprise more than 250 valid species (Archidona-Yuste *et al*., 2016a,b; Zhao *et al*., 2017; Mirzaie Fouladvand *et al*., 2019; Jahanshahi Afshar *et al*., 2019; Cai *et al*., 2020; Fadakar *et al*., 2021), but currently only 19 have been reported from Nigeria, including *X. basiri* Siddiqi, 1959, *X. bergeri* Luc, 1973, *X. brasiliense* Lordello, 1951, *X. brevistylus* Jairajpuri, 1982, *X. cavenessi* Luc, 1973, *X. dihysterum* Lamberti *et al*., 1995, *X. elongatum* Schuurmans Stekhoven & Teunissen, 1938, *X. fatikae* Bos & Loof, 1984, *X. longicaudatum* (Luc, 1961) Luc & Hunt, 1978, *X. majus* Bos & Loof, 1984, *X. manubriatum* Luc, 1975, *X. nigeriense* (Luc, 1961) Coomans *et al*., 1999, *X*, *oryzae* Bos & Loof, 1984, *X. oxycaudatum* Lamberti & Bleve-Zacheo, 1979, *X. paritaliae* Loof & Sharma, 1979, *X. radicicola* Goodey, 1936, *X. rotundatum* Schuurmans Stekhoven & Teunissen, 1938, *X. savanicola* Luc & Southey, 1980, and *X. tarjani* Luc, 1975 (Cohn and Sher, 1972; Bos and Loof, 1984), among which 8 have been molecularly identified, viz. *X. basiri*, *X. brasiliense*, *X. elongatum*, *X. longicaudatum*, *X. oxycaudatum*, *X. paritaliae*, *X. radicicola*, and *X. savanicola* (Chen *et al*., 2005; He *et al*., 2005; Oliveira *et al*., 2005). During recent nematode sampling from a kola nut (*Cola* sp.) area in southern Nigeria, a dagger nematode population on the non-*americanum*-group of *Xiphinema* was detected, resembling *X. ifacolum* Luc, 1961. Therefore, the objective of the present study was to provide an accurate identification of the *Xiphinema* species detected in southern Nigeria by an integrative approach of morphological and molecular characterization by using the sequences of D2-D3 expansion segments of 28S rDNA and partial mitochondrial *COI* gene.

Soil samples containing a population of dagger nematodes resembling *X. ifacolum* were collected at a depth of 5 cm to 30 cm from the rhizosphere of kola nut (*Cola* sp.) trees in Litaye village, Ondo State, southern Nigeria. Nematodes were extracted from soil by a modified sieving and decanting method (Brown and Boag, 1988). Extracted specimens were processed to glycerol and mounted on permanent slides (Hooper, 1986). The light micrographs and measurements of the nematode population, including the main diagnostic characteristics (i.e., de Man indices, body length, odontostyle length, lip region, tail shape, amphid shape, and oral aperture-guiding ring) were performed using a Leica DM6 compound microscope with a Leica DFC7000 T digital camera (Leica Biosystems, Wezlar, Germany). All other abbreviations used were as defined by Jairajpuri and Ahmad (1992).

For molecular analysis, specimens were preserved in DESS (Yoder *et al*., 2006), and DNA was extracted from single individuals as described by Archidona-Yuste *et al*. (2016a). Primers and polymerase chain reaction conditions used in this research were according to De Ley *et al*. (1999) and Lazarova *et al*. (2006). Single amplicons of *ca* 800 bp and 400 bp were obtained and sequenced for D2-D3 and *COI* regions, respectively. The newly obtained sequences were deposited into the GenBank database under the accession numbers OM777137-OM777138, OM758274-OM758275. Voucher specimens of this species have been deposited in the nematode collection of Institute for Sustainable Agriculture, IAS-CSIC, Córdoba, Spain.

Soil samples from the rhizosphere of kola nut (*Cola* sp.) trees in southern Nigeria yielded a *Xiphinema* population, including a moderate density (5–8 dagger nematodes/500 cm^3^ of soil) resembling *X. ifacolum*. The identity of the recovered population was further corroborated by molecular markers, including 28S rRNA and *COI* sequences, which were between 99.2% and 99.4% (differing in 3 indels, 2 gaps) identical in D2-D3 marker with a population of the same species from Sri Lanka (MH012181-MH012182), and between 96.2% and 96.3% (differing in 14 indels) identical in *COI* marker with the aforementioned population (MH013395-MH013396) (Susulovska *et al*., 2018). Although only two populations of this species have been sequenced from Sri Lanka and this one from Nigeria, intraspecific diversity in D2-D3 and *COI* follows the same pattern as other *Xiphinema* non-*americanum*-group species (Palomares-Rius *et al*., 2017). Maximum intraspecific distances for D2-D3 and *COI* sequences showed a higher molecular variability associated with *COI* than with D2-D3, establishing optimal barcoding differences of 2.87% and 6.36%, respectively (Palomares-Rius *et al*., 2017). Since the identity of this species has been confirmed by D2-D3 28S rRNA and the *COI* genes, no further studies than morphometrics were developed on this Nigerian population.

To our knowledge, this is the first report of *X. ifacolum* from Nigeria that is the geographically closest record to its type locality from Guinea (Luc, 1961). Morphological characters and morphometrics (Fig. 1, Table 1) of the Nigerian population agree with those of the type population and other populations from Brazil, Cameroon, Liberia, São Tomé and Príncipe, and Sri Lanka (Luc, 1961; Loof & Sharma, 1979; Rashid *et al*., 1986; Oliveira *et al*., 2003; Sakwe & Coomans, 1993; Lamberti *et al*., 1992; Susulovska *et al*., 2018).

**Figure 1:**
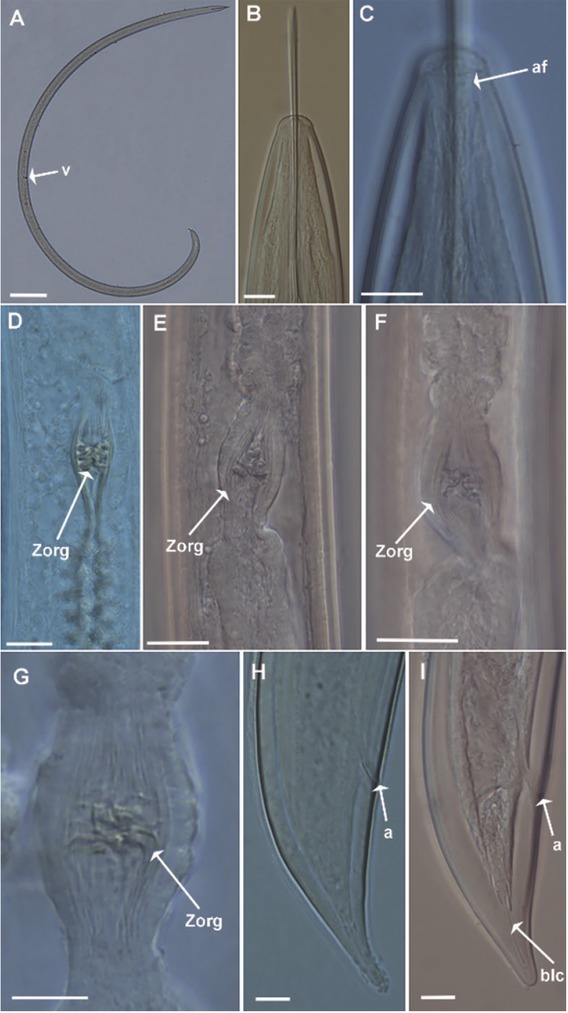
Light micrographs of *Xiphinema ifacolum* Luc, 1961 from Nigeria (A–I). (A) Whole female, (B,C) Female lip region showing amphidial fovea, (D–G) Detail of Z-organ in uterus, (H–I) Female tail regions. *Indicators*: af = amphidial fovea; a = anus; Zorg = Z-organ. (Scale bars: A = 200 μm; B, C, G–I = 10 μm; D–F = 20 μm).

**Table 1:** Morphometrics of *Xiphinema ifacolum* Luc, 1961 from Nigeria. All measurements are in micrometer, with the exception of L, which is expressed in millimeter; and all values take the form: mean ± s.d. (range).

Character/ratios^†^	Females
n	5
L (mm)	3.5 ± 0.18 (3.17–3.60)
a	58.6 ± 2.5 (54.6–60.6)
b	8.2 ± 0.5 (7.4–8.6)
c	56.1 ± 3.5 (51.9–60.9)
c′	1.7 ± 0.1 (1.6–1.9)
d^‡^	7.9 ± 1.5 (6.5–10.4)
d′^⚔^	3.1 ± 0.2 (2.8–3.3)
V	51.6 ± 1.4 (50.4–53.9)
G1	11.2 ± 0.6 (10.7–11.8)
G2	11.0 ± 0.7 (10.4–11.7)
Odontostyle length	125.6 ± 4.0 (121–131)
Odontophore length	69.5 ± 1.2 (68–71)
Total stylet length	195.1 ± 4.2 (190.0–201.5)
Anterior end to guide ring	94.6 ± 17.3 (84–125)
Tail length	62.2 ± 2.4 (59–65)
Hyaline part of tail length	19.8 ± 1.9 (17–21)
Body width at level of:	
Lip region	12.0 ± 0.7 (11–13)
Guide ring	36.8 ± 0.8 (36–38)
Anus	36.1 ± 1.9 (34.5–39.0)

† Abbreviations are defined in Jairajpuri and Ahmad (1992) ^‡^d = anterior to guide ring/body width at lip region (Brown *et al*., 1994) ^⚔^d′ = body width at guide ring/body width at lip region (Brown *et al*., 1994)

The Nigerian population of *X. ifacolum* is characterized by a moderate body length, a broadly rounded lip region that is slightly offset from body contour, a robust odontostyle and odontophore, and a double guiding ring (Fig. 1). The female reproductive system is didelphic-amphidelphic, with a tripartite uterus composed of short *pars dilatata uteri* followed by a well-developed Z-organ, comprising three to five sclerotized bodies of variable shape (from round to star-shaped) and size, and a tubular region devoid of spiniform structures (Fig. 1). The tail is a short, convex conoid, ventrally curved with a fine canal (“blind canal”), and surrounded proximately by a fine sleeve. According to the polytomous key proposed by Loof and Luc (1990), and as supplemented by the researches of Loof and Luc (1993), Loof *et al*. (1996) and Peraza-Padilla *et al*. (2017), the Nigerian population has the following specific a-numeric codes (codes in parentheses are exceptions): A4 - B1 - C3 - D4 - E6 - F34 - G2(3) - H2 - I3 - J3 - K2 – L1.

The present study indicates that there has been an increase in the number of *Xiphinema* species in Nigeria, and additionally reveals the presence of this genus of dagger nematode in the area of the Guinean golf, confirming that its distribution extends to tropical climate conditions (Luc, 1961; Loof & Sharma, 1979; Rashid *et al*., 1986; Oliveira *et al*., 2003; Sakwe & Coomans, 1993; Lamberti *et al*., 1992; Susulovska *et al*., 2018).
